# On a New Method of Operating for the Radical Cure of Hernia

**Published:** 1841-05

**Authors:** 


*On a new method of Operating for the radical cure of Hernia.* By M. Velpeau.—The author of this memoir, after examining at length the various means hitherto employed for the cure of hernia, states that he finds none of them at all efficacious; he therefore determined to try another method. Convinced from repeated experiments that injections with tincture of iodine produced in the serous membranes an adhesive inflammation, slight and scarcely dangerous, he attempted the radical cure of hernia by these injections. Three patients were operated on by him in the years 1836 and 1837, but the difficulties of a first operation, and the vagueness of the results, still left him in doubt, and he afterwards employed a method in two cases consisting of three distinct steps: subcutaneous puncture, scarification of the interior of the sac, and compression. He took the idea of scarifications from the ancients, or rather from what he had himself advanced in the year 1832. Twice that year he had placed great confidence in them, and only discontinued their employment when he perceived that danger was likely to ensue to the patient from opening the sac. These dangers seemed no longer likely, when M. Guerin gave the hint of the possibility of penetrating to the bottom of the cavity, by a simple puncture of the integuments, and expressed in his memoir his desire of applying his subcutaneous incisions to the radical cure of hernia. M. Velpeau has performed the necessary scarifications in this manner. With regard to the compression, he applies it not only to the external abdominal ring, but over the internal ring, and in the inguinal canal itself. He employs the celebrated bandages of M. Fournier of Lempdes, which he states have more than once sufficed for the radical cure of hernia.

M. Velpeau successfully operated on a patient in the following manner: Having introduced the index-finger for some lines in depth into the external abdominal ring, he pushed the integuments before it, and passed a sort of lancet over the nail of this finger, pushing it obliquely as far as the iliac fossa; then withdrawing his finger from the ring, he brought the edge of the instrument against the iliac walls of the abdomen, which he supported with the other hand, and then scarified them in various directions, taking especial care to avoid the epigastric vessels. The lancet was then withdrawn at the exact spot where it had entered, and the operation was complete, only a few drops of blood having been lost. The patient was taken to bed, no accident occurred, the small wound cicatrized by the next day, the bandage of Founier was applied on the third day, and the man has since walked and conducted himself as if he had never been afflicted.

Encouraged by this result, the patient demanded a similar operation on another hernia. It was performed, and at the end of ten days he was convalescent. He has since continued quite well, and has remained as a cook in the hospital to show the radical cure to the profession.—*Ibid. From Bulletin General de Therapuetique.* 15 *et* 30 *Aout*, 1840. *Ibid.*

